# The Influence of Structured Prenatal Education on The Use of Specialist Lactation Counselling in Case of Difficulties

**DOI:** 10.34763/devperiodmed.20192304.216226

**Published:** 2021-01-29

**Authors:** Mariola Błachnio, Ewa Dmoch-Gajzlerska, Magdalena Bednarczyk

**Affiliations:** 1 Department of Gynecology and Obstetrics Didactics, Faculty of Health Sciences, Medical University of Warsaw, Warsaw Poland; 2 Neonatology and Neonatal Intensive Care Clinic, Institute of Mother and Child, Warsaw, Poland

**Keywords:** structured prenatal education, breastfeeding, human milk, puerperium, edukacja przedporodowa, karmienie piersią, laktacja, połóg

## Abstract

**Introduction:**

Breastfeeding is the optima/ way to nourish newborns and infants. According to the PTGHiŻDz, ESPGHAN and AAP, exclusive breastfeeding should be sought for the first 6 months of life.

**Aim:**

To analyse the relationship between participation in prenatal education and the frequency to intend and then continue breastfeeding in the first 6 months of a child's life. To assess the impact of participation in prenatal education on women's use of specialist lactation counselling.

**Materiał and methods:**

The study was conducted in the period from May 2013 to June 2014 at the Specialist Hospital of St. Sophia in Warsaw. ft included 333 women in the maternity ward. The study group consisted of 244 women who participated in structured prenatal education classes in group form. The control group numbered 89 women who did not participate in prenatal education either in one-to-one or group form. The first stage of the study was carried out among women 48 hours after childbirth. In the second stage of the study a questionnaire was sent to women in electronic form, not earlier than one day after the child was 6 months old.

**Results:**

The variables that differentiated the groups studied were: education, place of residence, professional activity before pregnancy and professional activity during pregnancy longer than 27 weeks. The intention to take up exclusive breastfeeding expressed 48 hours after childbirth was declared by 94.7% of the women from the study group and 86.5% of the women from the control group (p=0.024). Breastfeeding was the most common difficulty reported by women during their stay in hospital (39.3% vs. 38.2% p=0.85) and during the first 6 months after childbirth (19.8% vs. 7.1°% p=0.11). In case of difficulties in the course of childbirth, the women from the study group who used specialised lactation counselling accounted for 12.7%. No woman from the control group benefited from this type of care.

**Conclusions:**

Participation in structured prenatal education is a factor influencing the frequency of intending to breastfeed. Women after a cycle of structured prenatal education classes are more likely to take advantage of lactation counselling at a specialist level. Structured prenatal education has no influence on the subjective assessment of women concerning the practical application of the knowledge concerning breastfeeding.

## Streszczenie

**Wsfęp:**

Karmienie piersiq jest optymalnym sposobem żywienia noworodków i niemowląt. Według PTGHiŻDz, ESPGHAN i AAP należy dążyć do wyłącznego karmienie piersią przez pierwszych 6 miesięcy życia dziecka.

**Cel pracy:**

Analiza związku pomiędzy uczestnictwem w edukacji przedporodowej, a częstością zamiaru karmienia piersią i jego kontynuacji w 6 miesiącu życia dziecka. Ocena wpływu uczestnictwa w edukacji przedporodowej na korzystanie przez kobiety ze specjalistycznego doradztwa laktacyjnego.

**Materiał i metody:**

Badanie przeprowadzono w okresie od maja 2013 roku do czerwca 2014 roku w Szpitalu Specjalistycznym św. Zofii w Warszawie. Objęto nim 333 kobiety przebywające na oddziale położniczym. Grupę badaną stanowiły 244 kobiety, które uczestniczyły w zajęciach edukacji przedporodowej w formie grupowej. Do grupy kontrolnej zakwalifikowano 89 kobiet które nie uczestniczyły w edukacji przedporodowej w formie indywidualnej lub grupowej. Pierwszy etap badania przeprowadzono wśród kobiet po 48h od urodzenia dziecka. W etapie drugim badania wysłano do kobiet kwestionariusz ankiety w formie elektronicznej nie wcześniej niż po dniu w którym dziecko ukończyło 6 miesiąc życia.

**Wyniki:**

Zmienne które różnicowały badane grupy to wykształcenie, miejsce zamieszkania, aktywność zawodowa przed ciążą oraz aktywność zawodowa w ciąży dłużej niż do 27 tygodnia ciąży. Zamiar wyłącznego karmienia piersią wyrażony po 48h od porodu dotyczył 94,7% kobiet z grupy badanej i 86,5% kobiet z grupy kontrolnej (p=0,024). Najczęstsza trudność jaką zgłosiły kobiety w trakcie pobytu w szpitalu (39,3% vs 38,2% p=0,85) i w trakcie 6 miesięcy po porodzie (19,8%vs 7,1% p=0,11) dotyczyła karmienia piersią. W przypadku wystąpienia trudności w przebiegu połogu kobiety z grupy badanej korzystały ze specjalistycznego poradnictwa laktacyjnego w przypadku 12,7% z nich (0,046). Żadna kobieta z grupy kontrolnej nie korzystała z tego rodzaju opieki.

**Wnioski:**

Uczestnictwo w edukacji przedporodowej jest czynnikiem wpływającym na częstość zamiaru karmienia piersią. Kobiety po cyklu zajęć edukacji przedporodowej częściej korzystają ze specjalistycznego poradnictwa laktacyjnego. Edukacja przedporodowa w formie zorganizowanej pozostaje bez wpływu na subiektywną ocenę kobiet dotyczącą wykorzystania zdobytej wiedzy w praktyce.

## Introduction

Breastfeeding is the optimal way to feed newborns and infants. For babies born before the due date and at term, both healthy and sick ones, it is the first choice in feeding. Natural breastfeeding has a health value for the baby as well as for the mother [1, 2, 3]. Infants who are breastfed are less likely to develop infectious diseases and contract such diseases less often. Moreover, they need fewer hospitalisations. They less often need medical counselling, and their mortality rates are lower [4]. The positive aspect of breastfeeding is its influence on the risk of many diseases in the mother, lower risk of complications in the puerperium, including lower tension, anxiety and incidence of postpartum depression [4]. The need to communicate the benefits of breastfeeding and breastfeeding behaviour to women is highlighted in the WHO/UNICEF Joint Paper under point 4 “10 steps to successful breastfeeding” [5]. According to the recommendations of the Polish and European Society of Paediatric Gastroenterology, Hepatology and Nutrition (PTGHiŻDz; ESPGHAN) and the American Academy of Pediatrics (AAP), it is necessary to strive for exclusive breastfeeding during the first 6 months of a child’s life [6, 7].

The strategy of WHO and many authors of world studies indicates a positive impact of women’s participation in prenatal education on the frequency and length of breastfeeding [5]. Polish research also shows that the impact of the project has been discussed. In the Kamianowska study, a significant influence on the frequency of the phenomenon was found in the group of mothers of premature babies after prenatal education [8].

The most beneficial intervention strategy to increase the frequency of breastfeeding and its duration is to combine pregnancy education, maternity support and lactation counselling in a womans living environment [5, 9,10].

Staff training is a practice that encourages natural feeding. Its desired effect is to update and standardise knowledge as well as to change attitudes and existing practices [11]. The expected impact of such courses was confirmed in the largest randomized trial conducted so far, i.e. The Promotion of Breastfeeding Intervention Trial (PROBIT) [12]. The effectiveness of lactation counselling has also been proven to extend the time and exclusivity of breastfeeding [13].

## Aim

1. To analyse the relationship between participation in prenatal education and the frequency of intending to breastfeed and continue in the first 6 months of a child’s life.

2. To assess the influence of participation in prenatal education on womens use of lactation counselling at specialist level.

## Material and methods

The Bioethics Committee at the Medical University of Warsaw did not raise any objections to how the study was conducted. The research material was collected from May 2013 to June 2014 at the Specialist Hospital of St. Sophia in Warsaw. The study included 333 women in the maternity ward. Based on the diagnostic survey method, the questionnaire for each stage of the study was constructed individually. The study group consisted of 244 women who participated in prenatal education classes in group form. The control group consisted of 89 women who did not participate in prenatal education in one-to-one or group form. The first stage of the study was carried out among women 48 hours after the birth of their child. All of them had given voluntary consent to participate in the study. In the second stage of the survey, the women interviewed filled in an electronic questionnaire which had been sent out not earlier than one day after the child turned 6 months old (6 m. of age). LimeSurvey Version 2.00+ Build 131206 was used for this purpose. Statistical analyses were performed with SPSS 17.0.0. Group comparisons were made using a non-parametric significance test - independence test chi2 and parametric t-Student test. During the analyses, the V-Cramer quota coefficient was also applied. For statistical reasoning the level of significance p<0.05 was assumed.

## Results

The groups of women studied differed in terms of education (p<0.001), place of residence (p<0.001), professional activity before pregnancy (p=0.012) and professional activity during pregnancy exceeding 27 weeks (p=0.006) Table I.

The problems (difficulties) reported by women during their stay in hospital with regard to breastfeeding, the postpartum period and during the care and nursing of an infant were equally frequent in the groups examined (p=0.797). The most frequent problems (difficulties) in both groups were related to breastfeeding (p=0.85) Table II. Preparation of women for the perinatal period during structured prenatal education did not influence the way of solving problems (difficulties) in the hospital (p=0.635) Table III. No differences were found between the frequency of problems (difficulties) in breastfeeding during 6 months after birth in the studied groups (p=0.11). In case they did occur, women from the study group more often used the lactation clinic (p=0,046) Table IV, Table V.

The women surveyed at the stage of the study conducted 6 months after childbirth comparably often specify the need to deepen their knowledge of physiology and the possible difficulties in the perinatal period (p=0.873) Table VI.Participation in structured prenatal education has influenced women’s intentions to breastfeed. Women from the study group surveyed in the first stage of the study (2 d. after) declare their intention to breastfeed significantly more often (p=0.024) Table VII.Structured prenatal education did not affect the frequency of natural feeding in the first 6 months of a child’s life (p=0.257). The frequency of exclusive breastfeeding in the study group and in the control group was found in 71.4% and 66.7% of respondents, respectively. (Table VIII). In a survey conducted 6 months after childbirth, women made a retrospective assessment of the use of knowledge acquired during structured prenatal education classes or on their own. Women from the study group significantly more often used their knowledge and skills in neonatal care (p=0.028) Table IX.

**Table I:** Characteristics of mothers–test/control group. Tabela I. Charakterystyka matek – grupa badana/kontrolna.

Characteristics of mothers Charakterystyka matek			Test group *Grupa Badana*	Control group *Grupa Kontrolna*	t- Student	
					Test value *Wartość testu*	Value p *Wartość p*
Age *Wiek* (years *lata*: M, Min/Max)			30.34 (21-40)	30.19 (20-40)	0.321	0.748
			1.55 (1-6)	1.65 (1-7)	-0.826	0.411
**Number of pregnancies** ***Liczba ciąż (n: M, Min.-Max.)***			**Test group** ***Grupa Badana***	**Control group** ***Grupa Kontrolna***	**2 test**	
					**Test value** ***Wartość testu***	**p value** ***Wartość p***
Education *Wykształcenie* (n)%	Primary education *Podstawowe*		-	(1)1.1	23.752	<0.001***
	Vocational	Zawodowe	(1)0.4	(2)2.2		
	Secondary Średnie		(14)5.7	(19)21.3		
	Higher *Wyższe*		(229)93.9	(67)75.3		
Place of residence *Miejsce zamieszkania* (n)%	Village *Wieś*		(12)4.9	(7)7.8%	25.238	<0.001^***^
	City<c>100,000 inhabitants *Miasto<c> 100 tys. mieszkańców*		(21)8.6	(27)30.3%		
	City<d>100,000 inhabitants *Miasto <d>100 tys. mieszkańców*		(211)86.5	(55)61.8%		
Marital status *Stan cywilny* (n)%	Married *Małżeństwo*		(206)84.4	(69)77.5	2.157	0.142
	Partner relationship *Zwiqzek partnerski*		(38)15.6	(20)22.5		
Number of deliveries *Rodność* (n)%	Primiparous *Pierworódki*		(184)75.3	(65)73	0.178	0.673
	Multiparous *Wieloródki*		(60)24.7	(24)27		
Professional activity before pregnancy *Aktywność zawodowaprzed ciqżq* (n)%			(236)96.7	(80)89.9	6.286	0.012^***^
Professional activity up to 27 weeks of pregnancy *Aktywność zawodowaw ciqży dłużej niżdo 27 tygodnia ciqży* (n)%			(86)35.8	(16)18.6	12.611	0.006^***^
Caesarean section *Cięcie cesarskie* (n)%			(45)18.3	(15)16.9	0.493	0.92

**Table II:** Type of problem (difficulty) arising during hospital stay (2 d. after) – test/control group. Tabela II. Rodzaj powstałego problemu (trudności) w trakcie pobytu w szpitalu (2 d. po) – grupa badana/kontrolna.

Problem (difficulty) duringstay in hospital (2 d. after) *Problem (trudność)* *w trakcie pobytuw szpitalu (2 d. po)*	Group *Grupa*				Chi-square *est chi-kwadrat*	
	Test *Badana*		Control Kontrolna			
	Number *Liczebność n=244*	%from column *z kolumny*	Number *Liczebność n=89*	%from column *z kolumny*	x^2^	P
Breastfeeding *Karmienie piersiq*	96	39.3%	34	38.2%	0.036	0.85
Infant care and nursing *Opieka i pielęgnacja dziecka*	8	3.3%	4	4.5%	0.277	0.598
Puerperium *Przebieg połogu*	10	4.10%	2	2.2%	0.643	0.423
Chi-square *Wartości testu chi-kwadrat*		x^2^=1.017; p=0.797				

**Table III:** Method of problem (difficulty) solving during hospital stay (2 d. after) –test/control group. Tabela III. Sposób rozwiązania powstałego problemu (trudności) w okresie pobytu w szpitalu (2 d. po) – grupa badana /kontrolna.

The way of solving the problem (difficulty) in hospital (2 d. after) *Sposób rozwiązania powstałego* *problemu (trudności) w szpitalu (2 d. po)*	Group *Grupa*				Chi-square *Test chi-kwadrat*	
	Test *Badana*		Control *Kontrolna*			
	Number *Liczebność n=244*	%from column *z kolumny*	Number *Liczebność n=89*	%from column *z kolumny*	x^2^	P
I solved the problem on my own *Samodzielnie rozwiązałam problem*	9	3.7%	2	2.2%	0.424	0.515
My midwife helped me *Pomogła mi położna*	94	38.5%	35	39.3%	0.018	0.894
I was helped by a neonatal midwife (nurse) *Pomogła mi położna(pielęgniarka) neonatologiczna*	20	8.2%	4	4.5%	1.337	0.248
I was helped by a gynecologist *Pomógł mi lekarz ginekolog*	12	4.9%	3	3.4%	0.363	0.547
Husband *Mąż*	1	0.4%	0	0.0%	-	-
Nobody helped me *Nikt mi nie pomógł*	1	0.4%	0	0.0%	-	-
Chi-square *Wartości testu chi-kwadrat*		x^2^=4,307; p=0,635				

**Table IV:** Type of problem (difficulty) during 6 months after birth – test/control group. Tabela IV. Rodzaj powstałego problemu (trudności) w trakcie 6 miesięcy po porodzie – grupa badana/kontrolna.

Problem (difficulty) during 6 months after birth *Problem (trudność)w trakcie 6 miesięcy po porodzie*	Group *Grupa*				Chi-square Test chi-kwadrat	
	Test *Badana*		Control Kontrolna			
	Number *Liczebność n=126*	%from column *z kolumny*	Number Liczebność n=28	%from column z kolumny	x^2^	P
Breastfeeding *Karmienie piersią*	25	19.8%	2	7.1%	2.555	0.11
Infant care and nursing *Opieka i pielęgnacja dziecka*	7	5.6%	0	0.0%	-	-
Puerperium *Przebieg połogu*	8	6.3%	3	10.7%	0.658	0.417
Chi-square *Wartości testu chi-kwadrat*		*X*^2^=6,994; p=0,072				

**Table V:** Method of solving the problem (difficulty) during 6 months after birth – test/control group. Tabela V. Sposób rozwiązania powstałego problemu (trudności) w trakcie 6 miesięcy po porodzie – grupa badana /kontrolna.

The way of solving the problem (difficulty) during 6 months after birth *Sposób rozwiązaniapowstałego problemu (trudności) w trakcie 6 miesięcy po porodzie*	Group *Grupa*				Chi-square *Test chi-kwadrat*	
	Test *Badana*		Control *Kontrolna*			
	Quantity *Liczebność n=126*	% from column *z kolumny*	Quantity *Liczebność n=28*	% from column *z kolumny*	*X*2	P
I solved the problem on my own *Samodzielnie rozwiązałam problem*	12	9.5%	1	3.6%	1.05	0.305
I saw a gynecologist *Byłam u lekarza ginekologa*	6	4.8%	3	10.7%	1.475	0.225
I was at the lactation clinic *Byłam w poradni laktacyjnej*	16	12.7%	0	0.0%	3.968	0.046***
I saw a pediatrician *Byłam u lekarza pediatry*	9	7.1%	1	3.6%	0.481	0.448
Advice from an environmentaland family midwife *Porada położnejśrodowiskowo - rodzinnej*	4	3.2%	0	0.0%	0.913	0.339
Chi-square *Wartości testu chi-kwadrat*		*X*2=10,643; p=0,063				

**Table VI:** Parental opinion on the need to deepen the knowledge on a selected topic (6 m. of age) – test/control group. Tabela VI. Opinia rodziców dotycząca potrzeby pogłębiania wiedzy z wybranej tematyki w szkole rodzenia i w nauce samodzielnej (6 m. ż. dz.) – grupa badana/kontrolna.

Parents' opinion on the need to improve their knowledge(6 m. of age) *Opinia rodzicówdotycząca potrzeby* *pogłębiania wiedzy (6 m. ż. dz.)*	Group *Grupa*				Chi-square *Test chi-kwadrat*	
	Test *Badana*		Control *Kontrolna*			
	Number *Liczebność n=126*	%from column *z kolumny*	Number Liczebność n=28	% from column z kolumny	*X*2	P
Course on the perinatal period *Tematyka prawidłowego przebieguokresu okołoporodowego*	92	73.0%	22	78.6%	0.368	0.544
Possible difficulties *Możliwe trudności*	106	84.1%	24	85.7%	0.044	0.834
Chi-square *Wartości testu chi-kwadrat*		*X*2=0.271; p=0.873				

**Table VII:** Women's intention to breastfeed a newborn (2 d. after) – test/control group. Tabela VII. Zamiar kobiet względem rodzaju karmienia noworodka (2 d. po) – grupa badana/kontrolna.

Women's intention to breastfeed a newborn baby (2 d. after) *Zamiar kobiet względem rodzajukarmienia noworodka (2 d. po)*		Group *Grupa*		Total *Ogółem*
		Test *Badana*	Control *Kontrolna*	
Breastfeeding *Karmienie piersiq*	Number *Liczebność*	231	77	308
	%from column *z kolumny*	94.7%	86.5%	92.5%
Mixed feeding - breast and modified milk *Karmienie mieszane - piersiqi mlekiem modyfikowanym*	Number *Liczebność*	12	12	24
	*z kolumny*%from column	4.9%	13.5%	7.2%
Feeding with modified milk *Karmienie modyfikowanymmlekiem*	Number *Liczebność*	1	0	1
	%from column *z kolumny*	0.4%	0.00%	0.30%
Total *Ogółem*	Number *Liczebność*	244	89	333
	%from column *z kolumny*	100%	100%	100%
Chi-square *Wartości testu chi-kwadrat*		*X*2=7.472; p=0.024***		
V-Cramer coefficient *Współczynnik V-Cramera*		V=0.150		

**Table VIII:** Type of newborn feeding (6 m. of age) – test/control group. Tabela VIII. Rodzaj karmienia noworodka (6 m. ż. dz.) – grupa badana/kontrolna.

Type of newborn feeding (6 m. ofage) *Rodzaj karmienia noworodka (6 m.ż. dz.)*		Group *Grupa*		Total *Ogółem*
		Test *Badana*	Control *Kontrolna*	
Breastfeeding *Karmienie piersiq*	Number *Liczebność*	90	18	108
	% from column *z kolumny*	71.4%	66.7%	70.6%
Mixed feeding - breast and modified milk *Karmienie mieszane - piersiq i mlekiem modyfikowanym*	Number *Liczebność*	14	6	20
	% from column *z kolumny*	11.1%	22.2%	13.1%
Feeding with modified milk *Karmienie mlekiem modyfikowanym*	Number *Liczebność*	22	3	25
	% from column *z kolumny*	17.5%	11.1%	16.3%
Total *Ogółem*	Quantity *Liczebność*	126	27	153
	% from column *z kolumny*	100%	100%	100%
Chi-square *Wartości testu chi-kwadrat*		*X*^2^=2.720; p=0.257		

**Table IX:** Women's practical application of knowledge acquired during structured prenatal education/independence (6 m. of age) – study/control group. Tabela IX. *Wykorzystanie przez kobiety* w *praktyce wiedzy pozyskanej podczas edukacji przedporodowej/samodzielnie (6 m. ż. dz.) – grupa badana/kontrolna*.

Women's use of the knowledge acquired by women (6 m. of age) *Wykorzystanie przez kobiety pozyskanej wiedzy (6 m. i. dz.)*	Group *Grupa*				Chi-square *Test chi-kwadrat*	
	Test *Badana*		Control *Kontrolna*			
	Number *Liczebnośćn=126*	%from column *z kolumny*	Number *Liczebnośćn=28*	%from column *z kolumny*	*X* ^2^	p
Puerperium *Okres połogu*	116	*92.1%*	26	92.9%	0.02	0.887
Breastfeeding *Karmienie piersią*	118	93.7%	26	92.9%	0.024	0.877
During infant care and nursing *Opieka i pielęgnacja dziecka*	125	99.2%	26	92.9%	4.835	0.028***

## Discussion

In the general population, information regarding infant feeding during the first 6 months of life is as follows: 4 - 18% of women feed their children exclusively with mothers milk, while 17 - 37% ( n=1679, n=486, n=795) provide mixed feeding [ 14-16]. The report of the Centre for Lactation Science (CNoL), “Breastfeeding in Poland” of 2015 (n=736), shows that after birth, 98% newborns are breastfed and 35.2% women breastfed a child up to 6 months of age [17]. According to other Polish studies, a similar percentage of women initiate breastfeeding equally frequently [13, 14]. In the United States of America, in a survey conducted among 1160 women with children 6 months of age, 24% of the mothers relied exclusively on breastfeeding. [18]. At the same time in Turkey breastfeeding was conducted by 36.5% of women who had broadened their knowledge of breastfeeding and 16.7% who had not (p<0.05) [19]. The Su study (Singapore) showed this dependence and confirmed the validity of combined education and practical training after childbirth in increasing the chances of exclusive breastfeeding [9].

In the Cochrane review of 2016, analysis was conducted of 20 studies, conducted among 9,789 women. The research was done in highly developed countries like: Australia, the USA, Canada. Advice from relatives, education organised during pregnancy or lactation consultations did not seem to increase the number of women initiating breastfeeding or its duration. However, some of the larger studies provide scientific evidence that education can be helpful [20].

Comparing the results with those of other authors, a significantly higher percentage of women engaged in exclusive breastfeeding in the first 6 months of childrens life in both groups analysed in the present study (71.4% vs. 66.7%). The “10 steps to successful breastfeeding” programme implemented in the hospital unit in which the study was conducted, which has been certified by the hospital since 1994 as a “child-friendly hospital” and respect for the principles of the International Code of Marketing of Products Substituting Womens Milk, had a probable impact on the frequency of the phenomenon. Presumably, the access the women had to specialist lactation care (III degree) in the maternity ward, the involvement of the employees of the maternity ward and cyclical training of hospital staff in lactation have also had an impact. In the hospital ward, women with difficulties in breastfeeding, or puerperium during infant care and nursing were most frequently assisted by midwives in both groups (38.5% vs 39.3% p=0.894).

According to the report of Professor Królak-Olejnik (n=1679), the most frequent reason for discontinuing breastfeeding at the age of 2, 4, 6 months given by mothers was “insufficient maternal milk to feed the baby”. In that study, 4% of the mothers breastfeed exclusively during the six months of a child’s life, while the corresponding figure was 14% among women giving birth in “Child-friendly hospitals”, where this is the highest percentage observed [15]. Dutch and American researchers, in a study on 3994 women, found that 40% of them stopped breastfeeding due to subjectively insufficient milk [21]. In the CNoL Report of 2018, in the question about the most frequent breastfeeding difficulties asked of the respondents, the above statement was also noted as the most frequent [22].

In the vast majority of cases, parents from both the groups examined express the need to deepen their knowledge of possible difficulties in the course of the perinatal period (84.1% vs. 85.7%). For some women, knowledge of the possible difficulties in the course of lactation may influence prevention and management. The most frequent reason for discontinuing breastfeeding described in the literature is not related to womens own choice, but to difficulties in breastfeeding, lack of sufficient information about the possibilities of lactation stimulation and insufficient support from the medical staff and their closest relatives [23]. Expanding the knowledge about possible difficulties and ways of solving them may influence the problem solving potential of those women who want to feed naturally.

In a study by other authors, structured prenatal education was a source of knowledge about breastfeeding for 17-44.67% of the women participating [24]. The level of knowledge in the general population of women depends on their level of education and their participation in structured prenatal education [8]. In this study, different forms of education also show differences among the women studied, and prove that participation in prenatal education classes resulted in a more frequent intention to breastfeed exclusively expressed after 2 days from childbirth (p=0.024). Strategies that increase the chance of success in breastfeeding include: access to lactation pumps, group activities during pregnancy, support from other women [25]. The factors influencing the length of feeding were also determined. These include: skin-to-skin contact, a rooming-in system, latching the newborn on immediately on first contact without rushing the first breastfeeding, while trying to ensure that it takes place at the time of the highest sucking readiness between the 20th and 50th minutes of life, certificate of the “Child-friendly hospital”, access to lactation counselling at general (II) and specialist level (III) [5, 15, 26]. It is important to look for factors that may influence breastfeeding length, as according to the report of the Childbirth with Dignity Foundation, although numerous factors influencing breastfeeding length are widely known and their use is protected in Poland by regulation, the relevant standards of conduct are not fully applied. In the Polish population, 21.6% of women do not receive assistance in the first latch on to the breast. They did not receive support from the staff, despite the fact that 17.8% of the women needed it. After caesarean section, only 50.3% of the women attempt to breastfeed in the postoperative room [27]. With the frequency of caesarean sections in Poland, underapplied standards may also contribute to the length of natural feeding.

Breastfeeding rates have a chance to improve when the medical community (midwife - lecturer, midwife in hospital and community care, doctor, neonatal nurse, physiotherapist, neurologopedist, psychologist and lactation counsellor) cooperate and ensure continuity of care [17].

In this study, 39.3% of women in the study group and 38.2% of women in the control group (p=0.85) reported difficulties in breastfeeding during their stay in hospital. In the general population, lactation difficulties are reported by 29.6-54% of women during their stay in the maternity ward, and they are significantly more common in primiparous women [14,28]. Klejewski demonstrated a higher level of knowledge of breastfeeding among women participating in prenatal school [29]. In the studies by Deluga and Żelazko women participating in structured prenatal education show greater theoretical knowledge of postpartum physiology and neonatal care and have no concerns about their skills [30, 31]. Soet argues that theoretical preparation strengthens a womans belief in her own abilities and skills, and consequently reduces stress [32]. In observations made among women in Italy (n=9004) who participated in antenatal education, it was found that the risk of a newborn being fed modified milk in hospital was halved [33].

Breastfeeding difficulties during the first 6 months after birth were found in this study among 19.8% of the women in the study group and 7.1% of the women in the control group. The study “Evaluation of the implementation of lactation practices within the existing standard of perinatal care and nutrition of children from birth to 12 months of age” shows that in hospitals where staff training in the practice of lactation took place, women are more often informed about the possibility of the visit of a midwife/ lactation counsellor's visit in the home environment [15]. However, according to Malańczuk, despite the efforts of hospital heads to train staff, the majority of women (75.1%) do not know the location of a lactation consultant and groups supporting breastfeeding women [34].

In the present study, it is shown that prenatal education influenced the frequency of seeking lactation advice at a specialist level during the first 6 consecutive months after childbirth (p=0.046). In the Cochrane review on breastfeeding support for healthy mothers of healthy children born at term, analysis was conducted of 100 randomized and control group studies among 83 246 women. It was found that additional support for women helps them to breastfeed longer [35]. The Mere wood study (n=108) found that lactation counselling had an effect among women who gave birth prematurely impacting breastfeeding length, a greater chance of breastfeeding undertaken by the mother (in any portion), and a greater chance of mainly breastfeeding the child. In studies by other authors, the care of a lactation counsellor is more effective compared to care that does not include counselling at a specialist level. A higher percentage of breastfeeding women was found after 1 week after childbirth, after 4 weeks, and also in the first 4 months of life [36].

## Conclusions

Participation in structured prenatal education is a factor influencing the frequency of intention to breastfeed.

Women after a cycle of structured prenatal education are more likely to take advantage of lactation counselling at a specialist level.

Structured prenatal education has no influence on the subjective assessment of women regarding the practical application of their knowledge on breastfeeding.
